# Leptomeningeal carcinomatosis in sinonasal NUT carcinoma: a case report and review of intracranial involvement

**DOI:** 10.3389/fonc.2026.1915873

**Published:** 2026-07-28

**Authors:** Francisco Manuel Rodríguez-Santiago, Silvia Sequero López, Antonio Jesús Láinez-Ramos-Bossini

**Affiliations:** 1 Department of Cardiology, Inter-hospital Cardiology Unit, San Cecilio University Hospital, Granada, Spain; 2 Cardiology Section, Department of Internal Medicine, Santa Ana Hospital, Motril, Granada, Spain; 3 FLG-NUT Foundation, Granada, Spain; 4 Department of Medical Oncology, San Cecilio University Hospital, Granada, Spain; 5 Instituto de Investigación Biosanitaria de Granada (ibs.GRANADA), Granada, Spain; 6 Department of Radiology, Virgen de las Nieves University Hospital, Granada, Spain; 7 Department of Human Anatomy and Embryology, School of Medicine, University of Granada, Granada, Spain

**Keywords:** BRD4-NUTM1, case report, head and neck neoplasms, leptomeningeal carcinomatosis, NUT carcinoma, sinonasal neoplasms

## Abstract

**Background:**

Sinonasal NUT carcinoma is a rare and highly aggressive malignancy, frequently diagnosed at advanced stages with poor prognosis. Intracranial spread is uncommon and, to our knowledge, leptomeningeal carcinomatosis has not been previously reported in sinonasal NUT carcinoma.

**Case description:**

We describe the clinical, radiological, histopathological, and molecular features of a 32-year-old woman with sinonasal NUT carcinoma who developed leptomeningeal carcinomatosis. The patient presented with a rapidly progressive nasoethmoidal mass with intracranial (dural) invasion. Diagnosis was confirmed by nuclear NUT immunoreactivity and identification of a *BRD4::NUTM1* fusion. Despite partial response to induction chemotherapy, the disease evolved with neurological deterioration and radiological findings consistent with contiguous intra-axial and diffuse leptomeningeal involvement, leading to death four months after diagnosis.

**Conclusions:**

Sinonasal NUT carcinoma with intracranial and meningeal extension may evolve to leptomeningeal carcinomatosis. Early diagnosis and multimodal treatment, including early surgery, may be crucial to improve prognosis.

## Introduction

NUT carcinoma (NC) is a rare, highly aggressive, poorly differentiated or undifferentiated carcinoma, characterized by rearrangements affecting the *NUTM1* gene, with the *BRD4::NUTM1* fusion being the most frequent alteration (≈ 70% of cases). Other described fusion partners include *BRD3*, *NSD3*, and several less common partners (1, 2). NC can affect numerous anatomical locations, predominantly involving midline structures such as the head and neck (2), where the sinonasal tract (nasal cavities and paranasal sinuses) is the most frequently involved location (3).

Due to its rarity and nonspecific clinical presentation, diagnostic errors or delays are common (4). Diagnosis at advanced stages with local invasion and metastasis is common, contributing to a poor prognosis with a median survival of 6.5 months (5). Diagnosis is established by diffuse nuclear immunoreactivity (≥ 50%) for the NUT protein using the C52B1 monoclonal antibody, or by demonstrating a *NUTM1* gene rearrangement through molecular techniques. These techniques, most commonly fluorescent *in situ* hybridization (FISH) and next-generation sequencing (NGS), provide prognostic information by enabling identification of the fusion gene and, in the case of NGS, aid in the differential diagnosis of very rare sarcomas harboring disease-specific *NUTM1* fusion partners (6).

Although the metastatic behavior of NC is well documented, intracranial and central nervous system (CNS) involvement is rare (7–10). To the best of our knowledge, leptomeningeal carcinomatosis (LC) has not been previously described in sinonasal NC.

This article aims to report the case of a 32-year-old woman with nasoethmoidal NC and clinical-radiological suspicion of LC, expanding the recognized spectrum of disease progression. In addition, a review of the literature on intracranial and CNS involvement in sinonasal NC is provided.

## Case description

**Figure 1:**
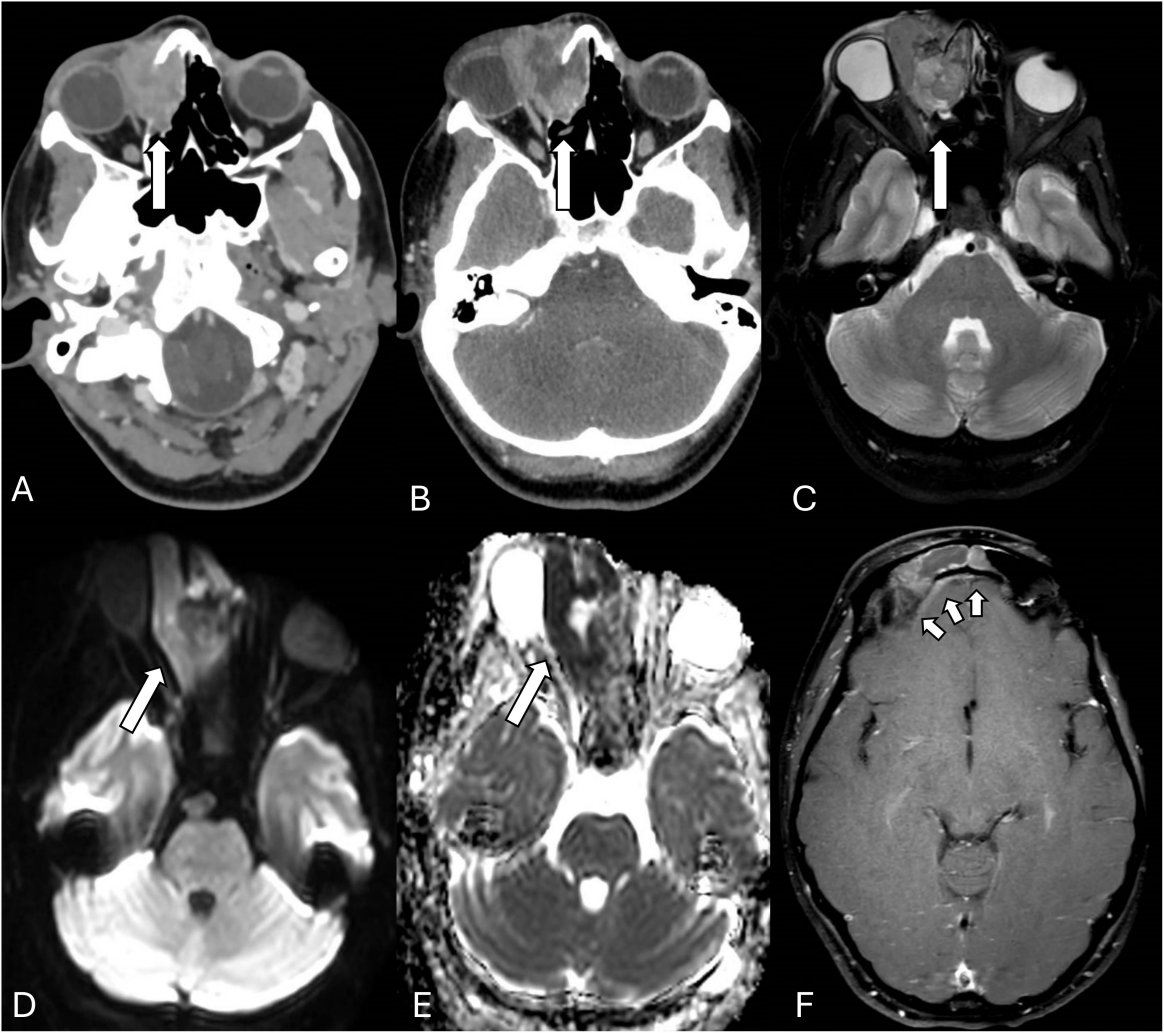
Contrast-enhanced computed tomography of the brain at initial imaging work-up **(A)** and 14 days later **(B)**. Right orbitoethmoidal mass (arrows) initially showing heterogeneous but mostly enhancing solid tissue with contiguous invasion of the medial aspect of the right orbit. Note the extensive osteolysis on the medial orbital wall, invasion of extraconal fat, and close contact with the medial rectus muscle with subtle sparing of the fat plane. **(B)** shows significant mass enlargement and extensive low-enhancing central areas that were not present in **(A)**, with increasing mass effect on the right ocular globe, which shows loss of sphericity and is displaced laterally. **(C-F)** Axial MR images two days after **(B)** [**(C)**, T2-weighted; **(D)**, diffusion-weighted image –b=1500-; **(E)**, ADC map; **(F)**, Post-contrast T1-weighted]. The mass shows heterogeneous but predominantly low T2 signal intensity, as well as high DWI signal and low ADC values consistent with significant diffusion restriction. **(F)** shows thickening and increased enhancement of the dura mater below the right frontal sinus, consistent with dural invasion [short arrows in **(F)**]. ADC, apparent diffusion coefficient; DWI, diffusion-weighted imaging; MR, magnetic resonance.

A 32-year-old woman with a medical history of bronchial asthma, migraine, and atopic dermatitis presented to the emergency department with a two-week history of sinusitis unresponsive to antibiotic therapy, associated with a mass at the superomedial angle of the right orbit, headache, and diplopia. Contrast-enhanced computed tomography (CECT) demonstrated a soft tissue mass centered on the right nasolacrimal duct, measuring 27 × 23 × 31 mm, with heterogeneous density, contrast enhancement, and intralesional cystic areas. The lesion extended to the nasal cavity, extraconal fat, and ethmoid air cells, with associated osteolysis of these structures and of the medial orbital wall (**Figure 1A**). An outpatient biopsy was performed under the initial suspicion of a mucocele.

After 14 days, the patient was readmitted due to severe pain and right eyelid edema preventing eye opening. A new CECT demonstrated significant enlargement of the orbitoethmoidal mass with a low-enhancing intralesional area (**Figure 1B**). She was admitted to hospital under suspicion of abscess formation. Given the rapid progression and the provisional histopathological report of an undifferentiated carcinoma, the diagnostic workup was completed with contrast-enhanced magnetic resonance imaging (MRI). Imaging revealed a markedly enlarging mass centered in the right nasoethmoidal region, extending to the ipsilateral orbit and maxillary sinus, as well as both frontal sinuses. The lesion exhibited T2 hypointensity, marked diffusion restriction, a central necrotic component, and moderate contrast enhancement with a low-enhancing central area. Extensive osteolysis and dural thickening with adjacent enhancement along the frontal sinuses were also noted, suggesting tumor infiltration (**Figures 1C–F**).

Five days later, the patient underwent 18F-fluorodeoxyglucose positron emission tomography (FDG PET-CT), which demonstrated intense hypermetabolic activity in the primary lesion (SUL_max 12.94) without evidence of macroscopic distant disease. Surgical resection was not recommended by the multidisciplinary tumor board given the locally advanced stage (cT4bN0M0, AJCC 8th edition), rapid tumor growth, aggressive clinical behavior, and lack of definitive histopathological characterization, raising concern for unfavorable tumor biology.

### Diagnostic assessment

To obtain a definitive histopathological characterization, the biopsy specimen was sent to a reference center. Following review of the initial biopsy specimen, the reference center issued a preliminary pathological impression of NC. However, the initial sample was insufficient for complete diagnostic evaluation, including immunohistochemistry (IHC) and molecular studies. Therefore, a second biopsy was performed during the diagnostic workup. Definitive diagnosis was established 18 days after the biopsy specimen was referred to the reference center. During this interval, rapid tumor progression was documented on contrast-enhanced MRI, which demonstrated a markedly enlarging mass measuring 40 × 56 × 75 mm, extending into the nasal cavity and cribriform plate, with supraorbital dural infiltration but without evidence of leptomeningeal or cerebral parenchymal enhancement. Subsequently, in the setting of clinical deterioration with refractory pain and facial paralysis, systemic chemotherapy (ChT) was initiated based on the preliminary pathological impression of NC and the patient’s rapidly progressive clinical course, before complete histopathological and molecular characterization became available. After the first ChT cycle, the diagnosis of NC was confirmed by IHC, which demonstrated nuclear NUT expression with a speckled pattern, absence of cytokeratins 8/18 and AE1-AE3, weak CD99 positivity, and focal vimentin and actin expression. FISH revealed *NUTM1* rearrangement. Subsequent NGS analysis identified a *BRD4::NUTM1* fusion involving *BRD4* exon 11 and *NUTM1* exon 3.

### Therapeutic intervention

**Figure 2:**
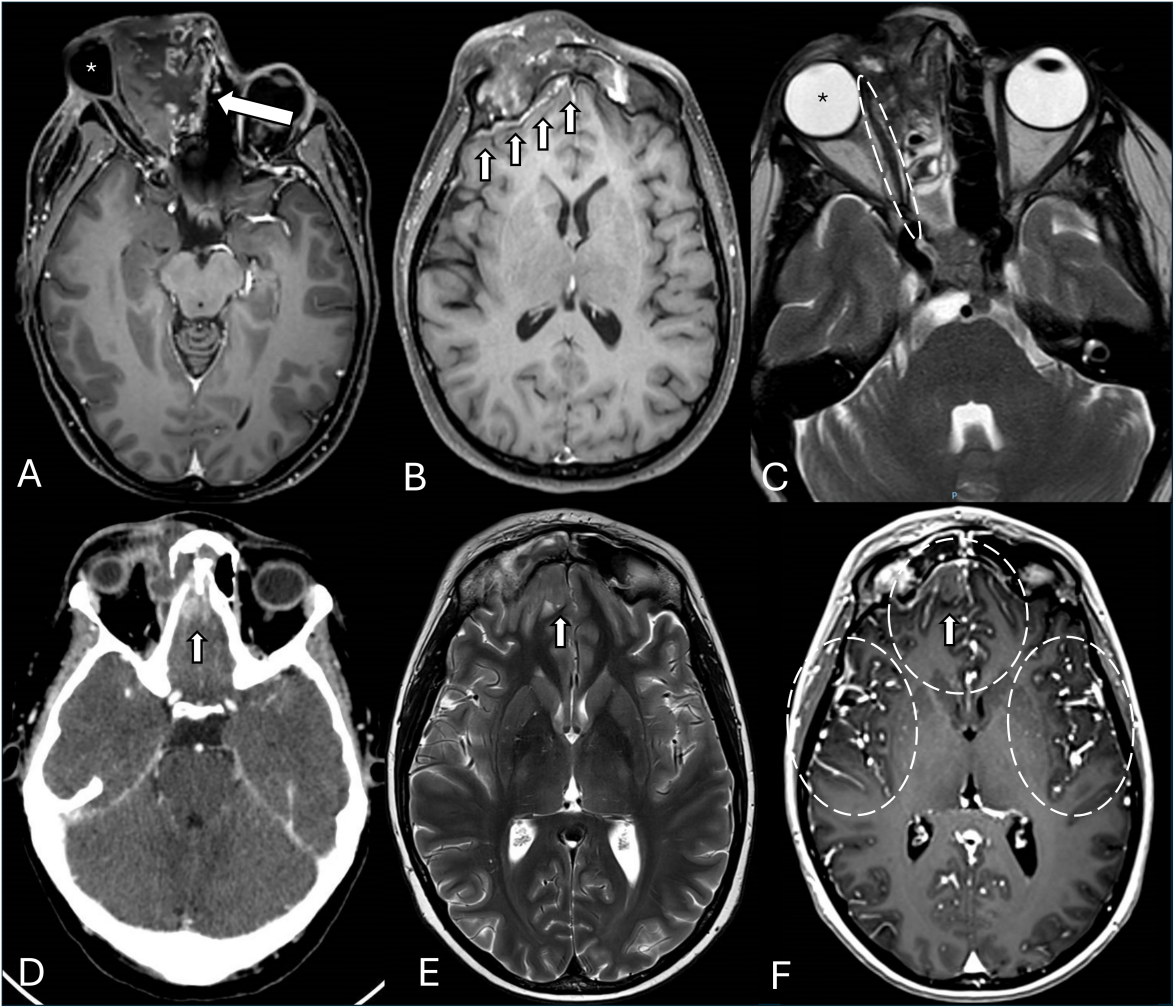
**(A, B)** Axial contrast-enhanced T1-weighted MR images at the start of chemotherapy showing significant enlargement and heterogeneous enhancement (low-enhancing areas consistent with extensive necrosis) of the right orbitoethmoidal mass (arrow), which exerts obvious mass effect on the right ocular globe [asterisk in **(A)**], being accompanied by dural thickening and hyperenhancement consistent with dural invasion [short arrows in **(B)**]. **(C)** Axial T2-weighted image at the end of chemotherapy showing a significant decrease in size of the mass, which is predominantly hypointense and confined to the ethmoid cells. Note the reduction in the mass effect on the right globe compared to A, with sparing of the fat plane of the right medial rectus muscle (dashed ellipsoid). **(D)** Axial contrast-enhanced brain CT at the time of seizure onset [9 days after **(C)**], showing a newly developed area of nodular enhancement at the anterior orbitofrontal region, consistent with intra-axial invasion (arrow). **(E, F)** Axial T2-weighted and contrast-enhanced T1-weighted MRI images [7 days after **(D)**] showing cortical thickening of the right straight gyrus with subtle subcortical edema (arrows). **(F)** shows thickening and enhancement of the dura mater in the right anterior orbitofrontal region, as well as scattered linear leptomeningeal enhancement and mild thickening, predominantly at the anterior orbitofrontal gyri and sylvian fissures (dashed ellipsoids), consistent with leptomeningeal carcinomatosis. CT, computed tomography; MR, magnetic resonance.

Systemic ChT with cisplatin, paclitaxel, and ifosfamide was administered every 2–3 weeks. After four cycles (8 weeks), follow-up MRI and FDG PET-CT showed partial remission, with tumor reduction to 23 × 36 × 29 mm, decreased intraorbital extension (**Figures 2A–C**), and reduced metabolic activity (SUL_max 5.45). Accordingly, surgical resection was planned.

### Follow-up and outcomes

However, 9 days after imaging reassessment demonstrating partial remission, and 13 days before the scheduled surgery, the patient experienced a generalized seizure. An emergency CECT was performed, demonstrating osteolysis of the cribriform plate and frontobasal enhancement suggestive of leptomeningeal infiltration, consistent with early intracranial progression (**Figure 2D**). Electroencephalography showed epileptiform activity in the left frontotemporal and central temporal regions. Levetiracetam was initiated.

In the following week, the patient developed severe headache, nausea, intractable vomiting, neck pain, behavioral changes, ataxia with gait impairment, and a new generalized seizure. Contrast-enhanced MRI showed stability of the nasoethmoidal mass with an extensive necrotic component but revealed contiguous intra-axial invasion of the right straight gyrus, characterized by cortical thickening, marked diffusion restriction, mild subcortical edema, and associated dural thickening and enhancement, together with scattered linear leptomeningeal enhancement involving the orbitofrontal regions (predominantly on the right), sylvian fissures, suprasellar and interpeduncular cisterns, cranial nerves (trigeminal and vestibulocochlear), and cerebellar folia (**Figures 2E, F**; **Supplementary Figures 1A–L**). These radiological findings were accompanied by worsening electroencephalographic abnormalities.

The characteristic distribution of these MRI findings, together with the patient’s rapidly progressive neurological deterioration and histologically confirmed NC, was considered highly consistent with probable LC. Clinically, behavioral changes and seizures coexisted with more diffuse neurological symptoms (e.g., severe headache, neck pain, intractable vomiting, ataxia), likely reflecting the concurrent presence of contiguous invasion of the right straight gyrus and diffuse leptomeningeal dissemination. However, the individual contribution of each process to the clinical presentation could not be determined.

**Figure 3:**
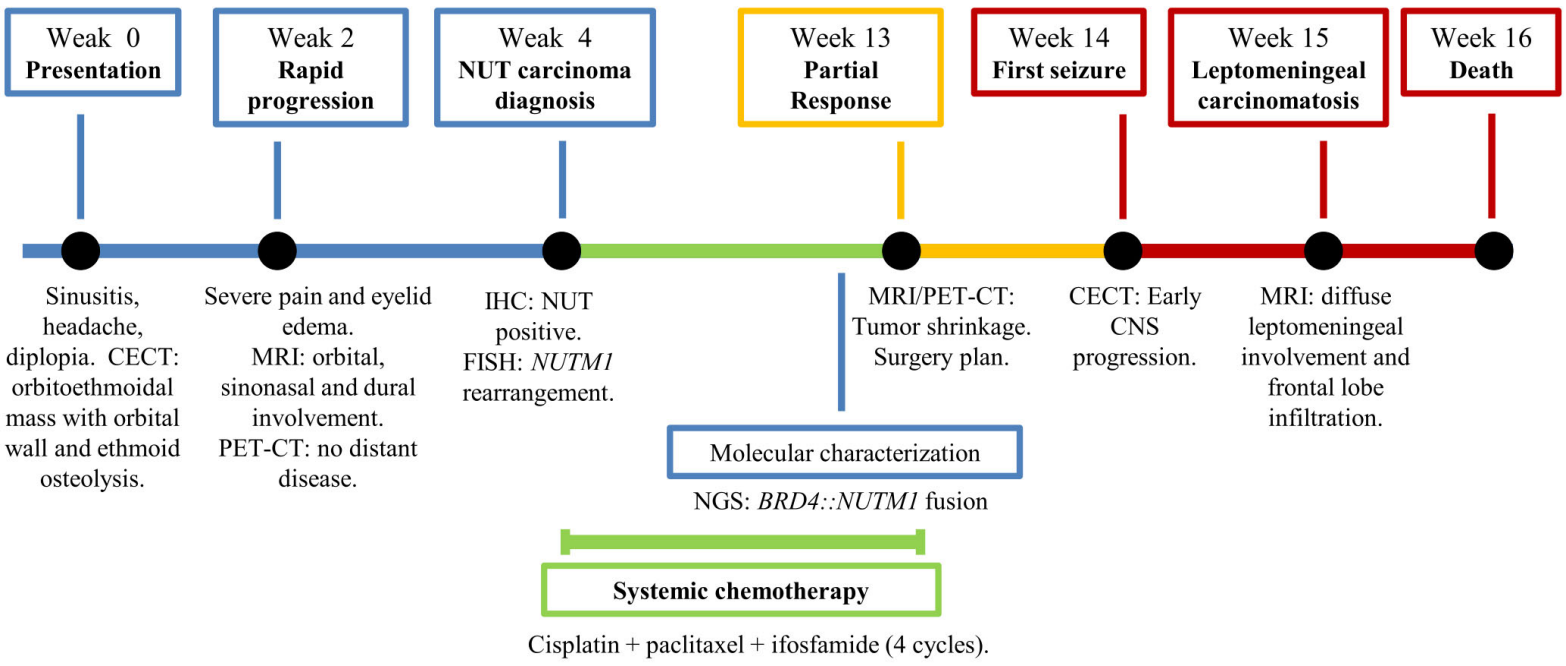
Clinical timeline of the patient’s disease course.

Cerebrospinal fluid (CSF) analysis was not performed because, in the setting of rapidly progressive neurological deterioration, poor overall clinical condition, and extensive intracranial disease, CSF examination was considered unlikely to provide additional diagnostic information that would alter therapeutic management. The patient succumbed to the disease three days after the diagnosis of probable LC and four months after the initial diagnosis. The patient’s clinical course is summarized in **Figure 3**.

## Discussion

### Diagnostic considerations

The diagnosis of sinonasal NC is particularly challenging due to its morphologic overlap with other poorly differentiated or undifferentiated sinonasal neoplasms, with initial misdiagnosis rates reported between 5% and 57% in the literature (7, 11). Although rare, the true prevalence of sinonasal NC remains unknown, with some reports suggesting that it accounts for approximately 12.5% of carcinomas of the sinonasal tract (3). This highlights the need for increased clinical awareness to recognize this entity, minimize diagnostic errors, and reduce underdiagnosis.

Histologically, NC typically consists of monomorphic small-to-medium cells with high mitotic activity and areas of necrosis, occasionally demonstrating abrupt focal or extensive keratinization. On IHC, it generally expresses pancytokeratins and squamous markers (p63, p40). In rare cases, as in the present case, these markers may be negative (12, 13).

Therefore, in rapidly growing sinonasal tumors with aggressive clinical behavior and poorly differentiated or undifferentiated morphology, NC should be considered in the differential diagnosis, and early exclusion by IHC using the monoclonal NUT antibody C52B1 is recommended (2). This case highlights the importance of early diagnosis, particularly in a disease with a time-dependent unfavorable clinical course, as it may significantly influence therapeutic strategies and improve prognosis.

### Patterns of invasion and dissemination

Sinonasal NC is characterized by extremely aggressive behavior, rapid growth, locoregional invasion, and early metastatic dissemination (14). Cervical lymph nodes represent the most frequent site of metastasis, followed by distant metastases to bone and lung, and less commonly, to the liver and CNS. Other reported metastatic sites include the spleen, adrenal glands, kidneys, pleura, pancreas, and thyroid (7–10). Ahmadian et al., in a systematic review of 45 patients with sinonasal NC, reported a high rate of distant metastatic dissemination (67%), present at diagnosis in 36% and during or after treatment in 31%. They also observed a predominance of advanced-stages disease (TNM stage III-IV in 95%), largely due to frequent locoregional involvement (7). Similarly, Baba et al., in a systematic radiologic review of 35 patients, described a highly invasive pattern characterized by destruction of adjacent structures, intraorbital extension (52.9%), and intracranial extension (29.4%) (8).

Intracranial and CNS involvement in sinonasal NC has been reported almost exclusively by contiguity, mainly through the skull base and, less frequently, via the orbit (7–10). This pattern of involvement is poorly characterized in the literature; therefore, a summary of published cases with intracranial and CNS involvement is presented in **Table 1**. Of the 18 reported cases, the primary tumor location was most commonly the ethmoid sinus (38.8%), followed by the nasal cavity (22.2%). Among the 16 cases in which the route of extension was specified, intracranial and CNS involvement occurred predominantly by contiguity through the skull base, except for one case with distant intraspinal metastasis without intracranial involvement. The most frequent intracranial involvement was parenchymal, followed by involvement of cavernous sinus, optic pathways, dura mater, and intradural space.

**Table 1:** Characteristics of sinonasal NC with intracranial and central nervous system (CNS) involvement.

Author (year)	Age/Sex	Tumor location	Route of extension	Intracranial involvement	Distant metastases	Pathologic confirmation	Treatment(s)	Overall survival (months)/Status at last follow-up
Hsieh et al. (15) (2011)	54/F	NC	SB	Parenchymal (bilateral frontal base and left inferior frontal lobe)	N/R	IHC +/FISH (BDR4::NUTM1)	CRT + Sx	N/R
Fang et al. (16) (2013)	55/M	N/R	ORB	Location N/R	None	IHC +	Sx + RT	40/AWD
	42/M	ES	ORB	Parenchymal (right frontal lobe)	None	IHC +/FISH (BRD4::NUTM1)	Neo RT + Sx + CRT	12/D
	50/M	FS	N/R	Parenchymal (location N/R)	None	IHC +/FISH (BDR4::NUTM1)	Sx	1/D
Stirnweiss et al. (17) (2015)	14/F	ES	SB	Parenchymal (right frontal lobe) and left optic nerve	None	IHC +/FISH, RT-PCR (BDR4::NUTM1)	Sx	3.2/D
Chan et al. (18) (2018)	48/M	NC	SB	Optic pathway	N/R	BRD4::NUTM1fusion	RT	N/R/D
Elkhatib et al. (19) (2019)	47/F	SS	SB	Cavernous sinus	Lung, retroperitoneal carcinomatosis, pancreas, duodenum	FISH (BRD4::NUTM1)	Sx + adjuvant CRT	5/D
Oliveira et al. (20) (2019)	42/M	MS	SB	Location N/R	Bone, lung	IHC +/NGS (BDR4::NUTM1)	CRT	11/D
Vakani et al. (21) (2020)	44/M	ES	SB	Cavernous sinus	N/R	IHC +	N/R	N/R
Sopfe et al. (22) (2021)	12/F	NC	SB	Location N/R	Bone	IHC +/FISH NUTM1	Neo CRT + Sx	40/ANED
Leeman et al. (23) (2021)	15/F	NC	SB	Location N/R	None	IHC +/FISH (BDR3::NUTM1)	CRT + Sx	34/ANED
Tosic et al. (24) (2021)	47/F	SS	SB	Intradural, suprasellar, cavernous sinus, internal carotid artery	Bone, lung	IHC +/NGS (BDR4::NUTM1)	Sx + CRT + IMx	4/D
Muramatsu et al. (25) (2022)	18/M	ES	SB	Optic pathway (left optic nerve) and epidural space	None	IHC +/FISH NUTM1 (no BRD4)	CRT + PBR	26/ANED
Huang et al. (26) (2022)	58/F	ES + SS	Systemic	No	Intraspinal	IHC +/NUTM1	Sx + CRT + DT + TKI + IMx + SDT	17/D
Wang et al. (9) (2023)	37/F	FS	SB	Location N/R	None	IHC +/FISH NUTM1	Sx + CRT	13/D
Caner et al. (27) (2023)	41/F	MS	ORB	Meningeal (dura mater)	Bone, liver	FISH and NGS (BRD4::NUTM1)	Neo CT + S + CRT	5.4/D
Holden et al. (28) (2025)^*^	43/M	ES	SB	Parenchymal (frontal lobe) and Meckel’s cave	N/R	IHC +	Sx	N/R/D
Baba et al. (8) (2025)	45/M	ES	SB/ORB	Location N/R	None	N/R	Sx + CRT	120/Alive
Current case	32/F	ES	SB/ORB	Meningeal (dura mater and leptomeningeal), parenchymal (gyrus rectus), leptomeningeal carcinomatosis	None	IHC +/FISH and NGS (BRD4::NUTM1)	CT	4/D

ANED, alive with no evidence of disease; AWD, alive with disease; CRT, chemoradiation therapy; CT, chemotherapy; D, dead; DT, deep thermotherapy; ES, ethmoid sinus; FISH, fluorescence *in situ* hybridization; IHC, immunohistochemistry; IMx, immunotherapy; M, male; MS, maxillary sinus; NC, nasal cavity; neo, neoadjuvant; NGS, next-generation sequencing; N/R, not reported; F, female; FS, frontal sinus; ORB, orbital; PBR: proton beam radiotherapy; RT, radiotherapy; RT-PCR, reverse transcription polymerase chain reaction; SB, skull base; SDT, sonodynamic therapy; SS, sphenoid sinus; Sx, surgery; TKI, tyrosine kinase inhibitor.

* Although classified as nasopharyngeal in the original report, the described clinical and radiologic findings are consistent with sinonasal involvement.

To the best of our knowledge, LC has not previously been reported as a metastatic pattern of sinonasal NC. In our patient, rapid neurological deterioration was accompanied by MRI findings highly suggestive of leptomeningeal involvement, including diffuse leptomeningeal enhancement affecting the cerebellar folia, cortical sulci, basal cisterns, and cranial nerves, together with contiguous intra-axial invasion of the right straight gyrus (29). Although leptomeningeal enhancement is not pathognomonic and may also occur in infectious or inflammatory disorders, these alternative diagnoses were considered unlikely because there were no clinical or laboratory features suggestive of CNS infection or inflammatory disease, whereas the characteristic imaging pattern developed in the setting of an aggressive histologically confirmed NC.

Despite the absence of CSF cytology and histopathological confirmation of LC, our case fulfilled the 2023 EANO–ESMO Clinical Practice Guideline criteria for probable leptomeningeal metastasis, based on histologically confirmed malignancy, compatible neurological manifestations, and characteristic contrast-enhanced MRI findings (29).

LC is defined as the spread of tumor cells within the leptomeninges and the subarachnoid space and represents an uncommon but devastating complication, occurring in up to 10% of patients with metastatic cancer during the course of their disease (29). Given that sinonasal carcinomas account for only 3–5% of head and neck cancers, LC in this tumor group is exceptional (30). According to the published literature, LC has been described in only a limited number of sinonasal carcinomas, including sinonasal squamous cell carcinoma (30), SMARCB1-deficient sinonasal carcinoma (31), sinonasal undifferentiated carcinoma (32), and sinonasal adenocarcinoma (33). Furthermore, Dagan et al. identified isolated leptomeningeal progression as the most common site of isolated distant metastasis in aggressive sinonasal carcinomas, highlighting this as an underrecognized pattern of disease progression (34). Our case therefore expands the spectrum of intracranial dissemination described in sinonasal NC.

### Therapeutic and prognostic implications

Treatment of NC is usually based on an aggressive multimodal approach combining surgery, ChT, and radiotherapy (RT); however, responses are often limited and not sustained. Systemic treatment remains an area of active therapeutic development. Given the *BRD4::NUTM1* fusion identified in our patient, bromodomain and extra-terminal (BET) inhibitors represent the most biologically rational targeted strategy by directly targeting the molecular driver of the disease. Although clinical responses have been reported, their durability has generally been limited. Immune checkpoint inhibitors have also demonstrated occasional responses, although current evidence remains limited to small series and case reports (6, 35). Overall, targeted therapies directed against the pathogenic mechanisms of NC represent a promising therapeutic approach, but their use currently remains largely restricted to clinical trials (36). In our patient, neither BET inhibitors nor immune checkpoint inhibitors could be administered because they were not available within an appropriate timeframe, and the patient experienced rapidly progressive neurological deterioration leading to death shortly after the diagnosis of probable LC.

Surgical resection with negative margins has been consistently associated with improved overall survival (OS) in sinonasal NC (7). When surgery is not feasible, ChT and RT constitute the main therapeutic alternatives, albeit with limited benefit due to rapid tumor progression (36).

In the present case, given the biological aggressiveness of the tumor and the initial lack of complete histopathological characterization, induction ChT was initiated, with a plan for salvage surgery followed by adjuvant RT and ChT. However, due to tumor progression with CNS involvement, the therapeutic strategy could not be completed, despite a partial response of the solid tumor mass. Lack of response to systemic therapy in sinonasal NC with neurovascular invasion, even in the presence of partial response at the primary tumor site, has been previously described (37). Ramesh et al., in a systematic review of 12 patients with sinonasal NC, reported that induction ChT with platinum-based regimens followed by surgery and adjuvant ChT could improve OS (38). Furthermore, ifosfamide-based ChT regimens, compared to platinum-based regimens, have been associated with longer progression-free survival (4). Regarding RT, its use in sinonasal NC has been associated with improved OS, particularly when doses ≥50 Gy are delivered (39).

Awareness that LC may occur as a complication of sinonasal NC provides an opportunity for early intervention through timely recognition of intracranial and meningeal involvement, allowing consideration of more aggressive treatment strategies, particularly early surgical intervention, in selected patients. This strategy could contribute to improved local control and potentially prevent or delay progression to LC, with relevant prognostic implications. As shown in **Table 1**, the median OS among cases with intracranial and CNS invasion was 12.5 months (range 1–120), with survival data available for 14 of the 18 patients. These outcomes contrast with the OS observed in the present case, which was 4 months from diagnosis, underscoring the negative impact of LC on clinical course.

## Conclusions

Sinonasal NC demonstrates highly aggressive biological behavior. This case highlights the potential for LC as a progressive complication in the setting of intracranial invasion, associated with particularly poor prognosis. Early diagnosis using IHC and timely identification of intracranial involvement may facilitate a multimodal approach, including early surgery in selected patients, with the potential to prevent or delay this severe complication.

## Data Availability

The original contributions presented in the study are included in the article. Additional patient-level data are not publicly available due to privacy and confidentiality considerations. Further inquiries can be directed to the corresponding author.
